# Coupling Mechanism Analysis and Fabrication of Triaxial Gyroscopes in Monolithic MIMU

**DOI:** 10.3390/mi8100310

**Published:** 2017-10-16

**Authors:** Dunzhu Xia, Lei Xu

**Affiliations:** Key Laboratory of Micro-Inertial Instrument and Advanced Navigation Technology, Ministry of Education, School of Instrument Science and Engineering, Southeast University, Nanjing 210096, China; 220152697@seu.edu.cn

**Keywords:** fully decoupled, tri-axis gyroscope, micro inertial measurement unit (MIMU), decoupling beams, coupling stiffness, fabrication imperfection, fabrication process

## Abstract

A novel fully decoupled micro inertial measurement unit (MIMU) is presented in this paper. The proposed MIMU structure, mostly focusing on the gyroscope unit, is highly symmetrical and can be limited to an area of 10,000 μm × 10,000 μm. Both the tri-axis gyroscope and tri-axis accelerometer structures are fabricated on the same single silicon chip, which can differentially detect three axes’ angular velocities and linear accelerated velocities at the same time. By elaborately arranging different decoupling beams, anchors and sensing frames, the drive and sense modes of the tri-axis gyroscope are fully decoupled from each other. Several dynamic models, including decoupling beams with fabrication imperfections, are established for theoretical analysis. The numerical simulation made by MATLAB shows the structural decoupling of three sense modes, and indicates that the key decoupling beams, which affect the quadrature error, can be improved in design. The whole fabrication process, including silicon on glass (SOG) process, dry/wet etching as well as the methods for improving the fabrication quality, is then shown. Experiments for mode frequency and quality factors of four modes (drive, yaw, pitch and roll) have been performed, and are found to be 455 (6950.2 Hz), 66 (7054.4 Hz), 109 (7034.2 Hz) and 107 (7040.5 Hz) respectively. The analysis and experiment both prove that this novel MIMU has the potential value of further intensive investigation.

## 1. Introduction

Micro-electro-mechanical systems (MEMS) technology has now drawn tremendous attention in recent years due to booming market needs, especially in the automotive industry, navigation systems and consumer electronics products [1,2]. The motion and position of an object in space can be accurately mapped through the use of a ten degree-of-freedom (10-DOF) sensing microsystem comprising a three-axis magnetometer, a three-axis gyroscope, a three-axis accelerometer and a barometer [3]. MEMS accelerometers are used in many fields: from automotive (air-bag sensor, rollover detection sensor, etc.) to mobile phone applications. Micromachined gyroscopes, likewise, are at present in a rapidly developing state [4]. However, the integration of the MEMS accelerometer and gyroscope has lagged far behind the development of two individual units. The MEMS accelerometer and gyroscope are usually fabricated respectively on the two silicon chips, and independently assembled together as an inertial measurement unit (IMU) to sense the acceleration and angle that restricts the advantages of MEMS sensors in size, weight, cost, and power consumption [5]. Some companies such as Northrop (Hawthorne, CA, USA) and Endevco (San Juan Capistrano, CA, USA) have already developed the single axis accelerometer and gyroscope, and the three-axis accelerometer and gyroscope have also been developed, with the acceleration range for ±60 g and measurement angle for 3000°/s. However the stability is 15°/h and the random drift is 100°/h, which is unable to achieve practical application stage [6]. Virtus Advanced Sensors once developed a single chip 6-degrees-of-freedom (6-DOF) MEMS IMU, providing inertial measurements for use in soldier-worn systems but the two units are assembled by three single-axis accelerometers and three single-axis gyroscopes so that the whole IMU performance is ensured by integrating control circuit, and reducing the systematic stability [7]. Therefore, to enhance the systematic reliability, reduce the design costs and improve the precision of MEMS sensors, a single-chip-integrated micro inertial measurement unit (MIMU), integrating the function of accelerometer and gyroscope with six degrees of freedom, is becoming a hot topic for researchers in the past two decades, in which the gyroscope unit is actually the key component. 

The micro-gyroscope is one of the most important components of the MIMU. In 2002, Analog Devices reported a single-chip, surface micro-machined integrated gyroscope with atmospheric hermetic package [8]. Afterwards, the single-axis gyroscope, especially the *z*-axis gyroscope, attracted the attention of many researchers. An *x*-axis gyroscope with vertical drive and in-plane sensing was first proposed in 2005 [9]. During 2008–2011, researchers at Peking University developed a novel lateral drive and torsional z-sensing single-chip gyroscope, in order to lower the air damping and suppress mechanical coupling [10,11]. The tuning-fork gyroscope has become one of the main forms of micro-gyroscope in recent research [12,13].

Improving the mechanical sensitivity of the gyroscope, typically a spring-mass-damping system, is the core aspect for researchers [14]. Many methods can be taken for reference. Liu et al. optimized the shape of the sensing beam in a tuning fork gyroscope by a cellular automata approach, and increased the sensitivity by 5.93 times and the bandwidth by 40.7% [15]. Chouvion et al. analyzed the mode shape variation of a ring-based rate sensor induced by coupling beam modification by the ray tracing method [16].

The quadrature error of the gyroscope, defined as the direct coupling of drive mode to sense mode, is one of the most important error sources. Fabrication imperfection is recognized as the main cause of the quadrature error [17]. Although the drive and sense modes of a tuning-fork gyroscope are perfectly orthogonal to each other in design, inevitable fabrication imperfection can still lead to the non-orthogonality of the drive and sense modes and result in quadrature error [18]. As the phase difference between input angular rate (Coriolis) signal and quadrature signal is 90°, the quadrature error can be eliminated by phase-sensitive detection circuit. Many attempts have been performed to cancel the quadrature error so far. Compared with the electrostatic cancellation by applying differential DC potentials to the mechanical electrodes on the device [18], structural improvement is a much better approach that reduces the quadrature error from the source. Kashif et al. designed a novel 3-DOF non-resonant gyroscope having a 2-DOF drive-mode oscillator. The proposed gyroscope utilizes structurally decoupled active-passive mass configuration to achieve dynamic amplification of oscillation in 2-DOF drive-mode and even eliminates the need of mode matching [19]. Sonmezoglu et al. described a novel 3-axis MEMS gyroscope based on a single vibrating structure with a secondary “auxiliary” mass to induce motion in the proof mass, which significantly reduces the effect of coupling from drive mode to the sense mode [20]. Mochida et al. designed two types of micromachined gyroscopes with oscillation characteristic observed by a two-dimensional laser displacement meter [21]. Considering the current level of fabrication; mechanical decoupling by carefully designing the decoupling beams is a feasible method, since the imbalances in the mechanical beams are the dominant mechanical-error source for the quadrature error in micromachined gyroscope unit [17].

As for structure design, MIMU with tuning-fork structure is the most popular choice for its superiority in sensing differential Coriolis acceleration, which can effectively improve the sensitivity and linearity [13]. Since the mechanical coupling between the drive and sense modes of a gyroscope has a great impact on its performance, it is necessary to decouple the two modes. Moreover, the quadrature error introduced by fabrication imperfection will also affect the performance of the tri-axis gyroscope unit. The meticulously designed beams are the key components of the MIMU to transform the input inertial parameters to the mechanical deformation, as well as to decouple the drive and sense modes of the gyroscope unit and accelerometer unit [22].

The work reported in this paper mainly focuses on the micro gyroscope unit in the MIMU, as the design, analysis and fabrication of tri-axis micro gyroscope have much more complexity than those of tri-axis micro accelerometer. Mechanical coupling stiffness of diverse beams and the influence to stiffness of decoupled beams caused by non-idealities in the gyroscope are the emphasis of the present work. 

Since the previous work for the design of a fully decoupled tri-axis linear vibratory micro-gyroscope focused on mode matching without actual fabrication, error analysis and test, this paper puts emphasis on the analysis of quadrature error and mode coupling introduced by the actual fabrication process, and experimental verification is covered as well.

This paper is organized as follows. Section 2 introduces the working principle of the MIMU and mechanical coupling stiffness of various beams is calculated in detail. The causes of quadrature error introduced by fabrication imperfection are involved, as well. The simulation of non-idealities for the MIMU, of which the analysis between coupling stiffness matrix and mechanical sensitivity of drive-to-sense modes is in Section 3. The actual fabrication process of the MIMU and the causes of non-idealities introduced by fabrication imperfection are arranged in Section 4. Section 5 and Section 6 show the experimental analysis and conclusion of the present work.

## 2. Working Principle and Structure Design

**Figure 1:**
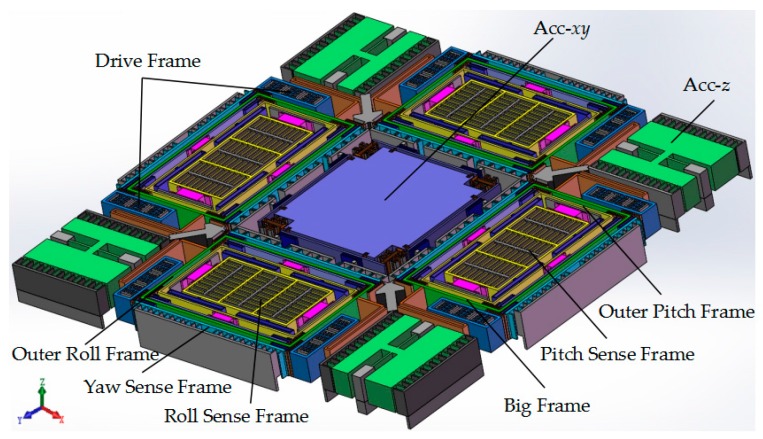
Schematic of the fully decoupled 6-DOF micro inertial measurement unit (MIMU).

The schematic of the presented MIMU, which contains the accelerometer unit and the gyroscope unit, is shown in Figure 1. The structural design and working principle of the MIMU can be described as follows, and this paper puts emphasis on the analysis of gyroscope unit.

For the accelerometer unit, it is a symmetrical structure with a central proof mass. The movable comb fingers that correspond to the fixed comb fingers of the sensing electrodes are solidly connected to the central mass via the crab-leg beams and U-shaped beams. The same four asymmetrical torsional masses are distributed in the four corners of the whole structure and are central circularly distributed with the central proof mass. 

Since the center proofmass is shared by both the accelerometer *x*/*y* units (Acc-*xy*), an acceleration x or y is applied onto the proofmass, which will lead to the change of the face-to-face resonant springs by level effect simultaneously and further changing the two resonant frequencies differently. The frequency split Δ*f* has a linear relationship with acceleration input approximately, so the acceleration in direction x or y can be detected.

The accelerometer *z*, comprised of four torsional masses (Acc-*z*), is designed for acceleration *z* detection. When an acceleration *z* is applied onto the unit, capacitance between the comb changes, which provides a measurement for the acceleration in direction *z*.

**Figure 2:**
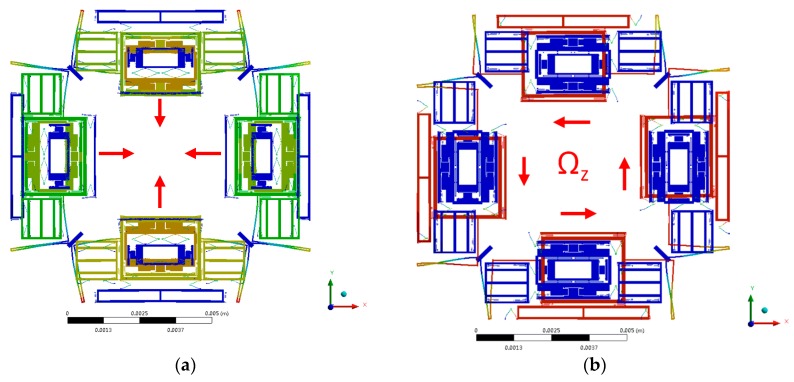

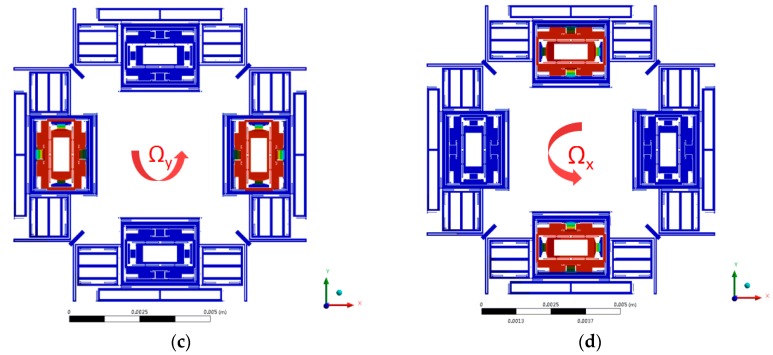
The modes of the gyroscope unit for the MIMU: (**a**) The Drive Mode; (**b**) The Yaw Mode (Ω*_z_*); (**c**) The Pitch Mode (Ω*_y_*); (**d**) The Roll Mode (Ω*_x_*).

The tri-axis gyroscope unit is a highly symmetric structure consisting of four Big Frames distributed at the periphery of the central proof mass. The four Big Frames are connected solidly via crab-leg beams, and various kinds of beams are designed elaborately to accomplish decoupling. The gyroscope unit is designed to have driving comb bonding with the drive beam that arranged on both sides of the Big Frame. The driving parts include the Drive Frame, Big Frame and the isolation masses (Outer Pitch/Roll Frame) for Pitch/Roll Modes. In Drive Mode, all the driving parts are driven to move together in the in-plane driving direction like a “beating heart”, when the driving voltage is applied on the fixed comb drive electrodes in the Drive Frame (Figure 2a). 

The Big Frame will generate an in-plane translational movement orthogonal with the driving direction as Yaw Mode, when an angular rate is applied on the gyroscope unit around the *z*-axis (Ω*_z_*), due to the Coriolis effect (Figure 2b). There are four Yaw Sense Frames distributed at the periphery of Big Frames.

The Pitch Mode is differentially detected by the Outer Pitch Frame and Pitch Sense Frame under angular velocity around *y*-axis (Ω*_y_*) shown in Figure 2c. The Outer Pitch Frame has 2-DOF in the in-plane driving direction and out-of-plane z-sensing direction. Upon the Outer Pitch Frame is steadily driven, when there is an angular velocity Ω*_y_* applied on the gyroscope, the Pitch Sense Frame will move together with the Outer Pitch Frame in *z* direction under the Coriolis effect. The sensing electrodes in Pitch/Roll Mode are designed to be comb fingers, which have different thickness in *z*-axis, so that the capacitance change is proportional to the *z*-axis movement caused by Coriolis force. 

The Roll Mode is differentially detected by the Outer Roll Frame and Roll Sense Frame under angular velocity around *x*-axis (Ω*_x_*) shown in Figure 2d. Its working principle is similar to the Pitch Mode. 

In this design, the fully decoupled mechanism depends on the elaborately arranged frames. As a result, the Drive Frame, Yaw Sense Frame and Sense Frame in Pitch/Roll Mode have only 1-DOF in their own drive or sense direction, respectively. The key point in the structure design lies in the decoupling of the in-plane movement in driving direction and the out-of-plane movement in Pitch/Roll Mode. Thus, the out-of-plane-decoupling beams with thinner width than the other structures adopted. They have relatively small stiffness in the *z*-axis direction and very big stiffness in the lateral axis direction, and thus can be used in the Pitch/Roll Mode to achieve drive-to-sense decoupling.

To improve the sensitivity of the gyroscope unit, it is essential to minimize the frequency split between the Drive Mode and three sense modes, since there are too many beams in this fully decoupled tri-axis gyroscope, which makes it complex and time consuming to realize the frequency matching process for four modes. Thus, some algorithms can be used for reference in order to quicken the process in mode matching. The particle swam optimization (PSO) can be adopted to optimize the beam dimensions and realize the mode matching in Drive Mode, Yaw Mode and Pitch/Roll Mode [23]. By setting a function of the resonant frequencies in all the drive and sense modes as the objective function, the mode matching process can be converted to the optimization of the established objective function with minimum value by PSO algorithm. Actually, the object function is the sum of quadratic difference between each working mode frequency and its corresponding object mode frequency.

Substituting all the dimensions of beams into the established model in ANSYS 14.5 (Canonsburg, PA, USA). The modal simulation results show that the resonant frequencies are 7009.8 Hz, 7067.4 Hz and 7089.2 Hz in Drive, Yaw and Pitch/Roll modes respectively.

## 3. Mechanical Coupling Stiffness Analysis

Fabrication imperfection is one of the most important sources in introducing quadrature-coupling error, which can directly influence the performance of the gyroscope unit. Even though the input angular rate is small, the quadrature error signal can be hundreds of times greater than the associated Coriolis signal and the output signal can be seriously affected [24]. A close-loop sense circuitry control system including the frequency-tuning control and phase and amplitude control can eliminate the quadrature error to some degree. However, it is more significant in device level to suppress the structural quadrature error than the subsequent signal processing. As for the structure of the present MIMU, various kinds of elaborately designed beams are adopted to achieve full decoupling. 

The Drive Frame is driven to move in the drive direction with the driving beams (D_1_, D_2_, D_3_, D_4_, D_1_’, D_2_’, D_3_’, D_4_’), when a driving voltage is applied on the drive electrodes. Under the participation of yaw coupling beams (Y_3_, Y_4_, Y_5_, Y_6_, Y_11_, Y_12_), the Big Frame will move together with the Drive Frames. The driving beams (D_5_, D_6_, D_7_, D_8_) will bring the yaw sense electrodes to move together with the Big Frame. Yaw sensing beams (Y_1_, Y_2_) will limit the yaw sense electrodes to have 2-DOF in-plane movement, when an angular rate in z-direction is applied on the gyroscope.

**Figure 3:**
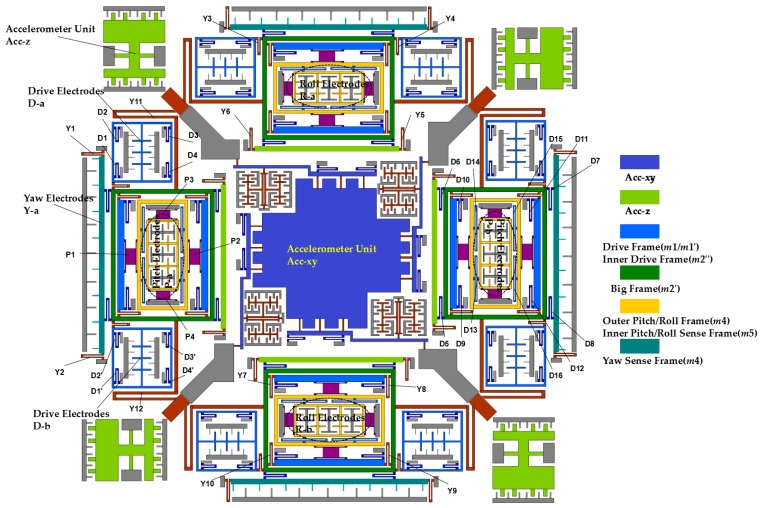
Schematic of the proposed Six-DOF MIMU with various decoupling beams.

The isolation mass for Pitch/Roll Modes and the Outer Frame in Pitch/Roll Modes will be driven to move together with the driving beams (D_9_, D_10_, D_11_, D_12_, D_13_, D_14_, D_15_, D_16_). When an angular rate in *x*/*y*-direction is applied on the gyroscope, under the participation of pitch/roll sensing beams (P_1_, P_2_, P_3_, P_4_), the Outer Frame in Pitch/Roll Modes has 2-DOF in the in-plane drive direction and out-of-plane sensing direction. The schematic diagrams for the gyroscope and various beams are shown in Figure 3 and a summary of all the driving and sensing beams and frames mentioned above is shown in Table 1.

Since all the drive and sense movements are transferred by various kinds of beams, it is necessary to establish the stiffness matrix for different beams of the gyroscope unit, and analyze the relationship between stiffness coupling coefficients and the structural parameters of the gyroscope unit. 

### 3.1. Mechanical Error Analysis

Once the Drive Mode is stably driven, a quasi-stable vibration will exist in the Sense Mode even without angular rate input, due to the coupling stiffness in structure. The response of the beam force introduced by the coupling stiffness, widely known as quadrature error, is always orthogonal to the Coriolis response. This is one of the main sources of zero-rate output (ZRO) of the micro-gyroscope [25]. The quadrature error, as well as cross-axis error introduced by fabrication process are greatly in connection with the ZRO, and ultimately influence the output of the MIMU.

Processing techniques such as the lithography process, silicon etching, bonding process etc. may introduce fabrication imperfection. Characterizing the non-idealities inherent in micro-gyroscope and accelerometers, including cross-axis sensitivity, offset bias and non-linearity, is quite necessary for industrial viability. The beams of the structure are relatively symmetrical in design, and the elastic system axes coincides to the proof mass axes in design, which makes the gyroscope unit structure fully decoupled. The in-plane and out-of-plane rotation of the elastic system axes introduced from fabrication has the maximum effect on mode decoupling.

Since all the beam geometries are identical, and the layout is totally symmetrical, the system stiffness matrix is diagonal because the off-diagonal can be cancelled out. Due to fabrication imperfection, the beams are not perfectly matched leading to a non-diagonal stiffness matrix (Equation (1)). The simplified elements of these off-diagonal terms can be expressed by the diagonal terms with some parameters, usually equivalent rotation [26].

(1) $$k_{system}=\left[\begin{array}{cc} k_{xx} & 0\\ 0 & k_{yy} \end{array}\right]\overset{\begin{array}{l} fabrication\\ imperfection \end{array}}{\to }k_{system}\prime =\left[\begin{array}{cc} k_{xx} & k_{xy}\\ k_{yx} & k_{yy} \end{array}\right]=\left[\begin{array}{cc} k_{xx} & f(k_{xx},k_{yy})\\ f(k_{xx},k_{yy}) & k_{yy} \end{array}\right]$$

where *k_system_* is the stiffness matrix of an ideal beam; *k_system_*’ is the stiffness matrix of beam with fabrication imperfection, which can be approximately expressed by diagonal terms.

**Figure 4:**
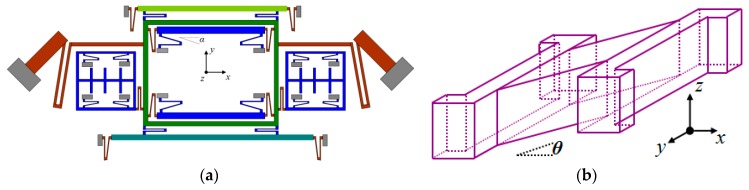
(**a**) In-plane fabrication error *α* of quadrature error for Drive Mode to Yaw Mode; (**b**) Out-of-plane fabrication error *θ* of quadrature error for Drive Mode to Pitch Mode.

In fabrication processing, especially in dry etching with SF_6_, the gas pressure difference between two U-shaped coupling beam arms can have the different etching rates. For specific performance, one of the two arms that are close to the anchor will be etched slower than that of the other arm as shown in Figure 4a, which is equivalent to having an additional angle α on the whole U-shaped beam and greatly affects the terms of the coupling stiffness (*k_xy_/k_yx_*). Due to the terms of the coupling stiffness introduced by fabrication imperfection, the whole gyroscope unit will have an undesired in-plane movement when the Drive Frames are steadily driven without angular velocity (Ω*_z_*) input, which will mostly affect the quadrature error for Drive Mode to Yaw Mode.

Analysis of fabrication imperfection for Trampoline decoupling beams can introduce an additional angle *θ* (Figure 4b) and lead to an out-of-plane movement in Pitch/Roll mode, when the Drive Frames are steadily driven without angular velocity (Ω*_y_*/Ω*_x_*) input.

Assuming the rotation angle is *α* and taking into consideration from the perspective of system stiffness, the transfer matrix can be expressed as:

(2) $$T_{R}=\left[\begin{array}{cc} \cos\alpha & \sin\alpha \\ -\sin\alpha & \cos\alpha \end{array}\right]$$

Substituting the ideal system stiffness matrix into Equation (2), the current equation can be calculated as:

(3) $$K_{Rtot}\prime =T_{R}^{T}K_{tot}T_{R}=\left[\begin{array}{cc} k_{xx}\cos^{2}\alpha +k_{yy}\sin^{2}\alpha & (k_{xx}-k_{yy})\frac{\sin2\alpha }{2}\\ (k_{xx}-k_{yy})\frac{\sin2\alpha }{2} & k_{yy}\cos^{2}\alpha +k_{xx}\sin^{2}\alpha \end{array}\right]$$

where *K_Rtot_’* is the current system stiffness matrix with rotation movement, *T_R_* is the transfer matrix and *K_tot_* is the ideal system stiffness matrix.

(4) $$K_{Rtot}\prime =\left[\begin{array}{cc} k_{xx} & k_{xy}^{\alpha }\\ k_{yx}^{\alpha } & k_{yy} \end{array}\right]=T_{R}^{T}K_{tot}T_{R}=\left[\begin{array}{cc} k_{xx} & (k_{xx}-k_{yy})\alpha \\ (k_{xx}-k_{yy})\alpha & k_{yy} \end{array}\right]$$

Actually, the contribution to quadrature error for Drive Mode to Yaw Mode is the summation of all the decoupling beams associated with the above two modes. Therefore, the equivalent matrix consists of driving beams in Drive Frame (D_1_, D_2_, D_3_, D_4_, D_1_’, D_2_’, D_3_’, D_4_’), yaw coupling beams with Big Frame (D_5_, D_6_, D_7_, D_8_, Y_3_, Y_4_, Y_5_, Y_6_, Y_11_, Y_12_) and yaw sensing beams in Yaw Frame (Y_1_, Y_2_). Take all the beams above into consideration, the equivalent matrix *k* is obviously the summation of each matrix for the coupling beams, the equation is shown below:

(5) $$k=\left[\begin{array}{cc} k_{xx} & k_{xy}\\ k_{yx} & k_{yy} \end{array}\right]=\left[\begin{array}{cc} 2\sum_{i=1}^{4}k_{ixx}^{\left(Di\right)}+\sum_{j=5}^{8}k_{jxx}^{\left(Dj\right)}+\sum_{l=1}^{12}k_{lxx}^{\left(Yl\right)} & 2\sum_{i=1}^{4}k_{ixy}^{\left(Di\right)}+\sum_{j=5}^{8}k_{jxy}^{\left(Dj\right)}+\sum_{l=1}^{12}k_{lxy}^{\left(Yl\right)}\\ 2\sum_{i=1}^{4}k_{iyx}^{\left(Di\right)}+\sum_{j=5}^{8}k_{jyx}^{\left(Dj\right)}+\sum_{l=1}^{12}k_{lyx}^{\left(Yl\right)} & 2\sum_{i=1}^{4}k_{iyy}^{\left(Di\right)}+\sum_{j=5}^{8}k_{jyy}^{\left(Dj\right)}+\sum_{l=1}^{12}k_{lyy}^{\left(Yl\right)} \end{array}\right]$$

where the *k_xx_* and *k_yy_* are the diagonal coefficients, while *k_xy_* and *k_yx_* are the off-diagonal coefficients in Equation (4).

Similarly, the equivalent matrix for Drive Mode to Pitch/Roll Mode consists of Pitch/Roll drive beams with Inner Drive Frame (D_9_, D_10_, D_11_, D_12_), Pitch/Roll coupling beams in Outer Pitch/Roll Frame (P_1_, P_2_) and pitch/roll sensing beams in Inner Pitch/Roll Frame (D_13_, D_14_, D_15_, D_16_, P_3_, P_4_). Table 2 shows the part of the structural dimensions with fabrication error, for example 0.1°.

To ensure the requirement of smooth vibration and acquire the needed working mode, the MIMU is completely symmetrical and center-symmetrically distributed in design, including the proof mass and coupling beams. As mentioned in the previous Section, due to the existence of fabrication error introduced by lithography process, silicon etching, bonding process etc., anisoelasticity of decoupling beams will be generated and affect the output of the MIMU.

Considering the dynamics equations of the MIMU under the existence of anisoelasticity of decoupling beams, two types of dynamic models can be confirmed: Drive Mode to Yaw Mode, Drive Mode to Pitch/Roll Mode. Table 3 shows the weight different frames in a quarter of the structure.

#### 3.1.1. Dynamics Analysis for Drive Mode to Yaw Mode

As is mentioned about the working principle of the MIMU in Section 2, different frames can be classified according to the degrees of freedom and establish the simplified dynamic models for analyzing the motion of the gyroscope in the drive direction.

**Figure 5:**
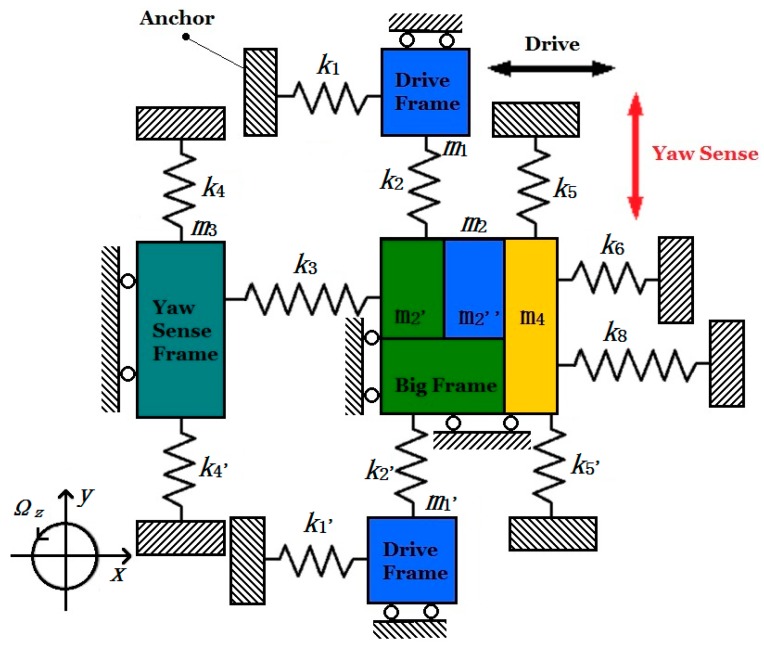
Simplified dynamic model for gyroscope in Drive Mode.

In Figure 5, *k*_1_ and *k*_1_’ are the summation of stiffnesses of U-shaped coupling beams D_1_, D_2_, D_3_, D_4_ and D_1_’, D_2_’, D_3_’, D_4_’; *k*_2_ and *k*_2_’ are the stiffness of decoupling beams Y_3_ and Y_4_; *k*_4_ and *k*_4_’ are the stiffness of coupling beams Y_1_, Y_2_, Y_5_ and Y_6_; *k*_3_ is summation of the stiffness of coupling beams D_7_ and D_8_ between Yaw Sense Frame and Big Frame; *k*_5_ and *k*_5_’ are the stiffness of crab-leg beams Y_11_ and Y_12_; *k*_6_ is the summation of the stiffness of coupling beams D_9_, D_10_, D_11_, D_12_; *k*_8_ is the summation of the stiffness of decoupling beams P_1_ and P_2_. All the labels of decoupling beams are shown in Figure 3. 

Moreover, *m*_1_ and *m*_1_’ represent the masses of Drive Frame, *m*_3_ represents the Yaw Sense Frame; *m*_2_’ and *m*_2_” represent the Big Frame and Inner Drive Frame respectively and *m*_4_ is the Outer Plane Frame (Pitch/Roll Mode). To simplify the dynamic model and equations, the related masses are classified in degrees of freedom: Drive Frames (*m*_1_ and *m*_1_’) and Inner Drive Frame (*m*_2_”) have one degree of freedom in direction *x*; Yaw Sense Frame (*m*_3_) has one degree of freedom in direction *y*. In particular, Big Frame (*m*_2_′) has two degrees of freedom in direction *x* and *y*, while Outer Plane Frame (*m*_2_”) has two degrees of freedom in direction *x* and *z*. Since *m*_2_’, *m*_2_” and *m*_4_ are driven together in direction *x*, when driving force is applied on the MIMU, so the three masses can be treated as a whole mass. 

One significant point of the MIMU is that both Yaw Mode and Pitch/Roll Mode share the same driving equation, since Big Frame (*m*_2_’), Inner Drive Frame (*m*_2_”) and Outer Pitch/Roll Frame (*m*_4_) move together by the driving force. The simplified dynamic equation in driving direction is shown below:

(6) $$(2m_{1}+m_{2}+m_{4})\overset{••}{x}+(2c_{1xx}+c_{3xx}+2c_{5xx}+c_{6xx}+c_{8xx})\overset{•}{x}+(2k_{1xx}+k_{3xx}+2k_{5xx}+k_{6xx}+k_{8xx})x+(2k_{5xy}+k_{6xy}+k_{8xy})y=F_{d}$$

**Figure 6:**
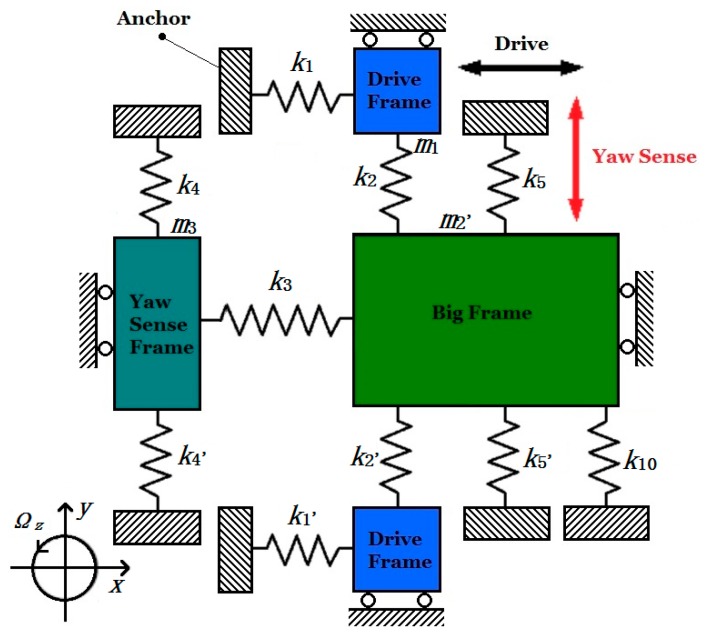
Simplified dynamic model for gyroscope in Yaw Mode.

In Yaw Mode, the Coriolis mass is the Big Frame (*m*_2_’) only with two degrees of freedom in direction *x* and *y*. So insulating the inner masses of the Big Frame, including the Inner Drive Frame (*m*_2_”) and Pitch/Roll Frames, the simplified dynamic model for Yaw Mode is shown in Figure 6.

In Figure 6, *k*_10_ represents the summation of U-shaped decoupling beams Y_7_, Y_8_, Y_9_ and Y_10_ (Figure 3). The other springs are the same in Figure 5. So the simplified dynamic equation in Yaw Sense direction is shown below:

(7) $$(m_{2}\prime +m_{3})\overset{••}{y}+(2c_{2yy}+c_{4yy}+c_{5yy}+c_{10yy})\overset{•}{y}+(2k_{2yy}+k_{4yy}+k_{5yy}+k_{10yy})y+(2k_{5yx}+k_{10yx})x=-2m_{2}\prime \Omega_{z}\overset{•}{x}$$

According to Equations (6) and (7), when the angular input Ω*_z_* = 0, owning to the existence of coupling stiffness *k_yx_*, coupling force *k_yx_x* generated by driving force in direction *x* will attach to the MIMU in direction y, and make the Big frame vibrate in the sense direction. 

Since the sensing displacement is far less than driving displacement, the coupling term (2*k*_5*xy*_ *+ k*_6*xy*_ *+ k*_8*xy*_)*y* can be neglected, and the dynamic equations of the gyroscope in drive direction x and sense direction y can be rewritten as follow:

(8) $$\begin{array}{l} \left[\begin{array}{cc} 2m_{1}+m_{2}+m_{4} & 0\\ 0 & m_{2}\prime +m_{3} \end{array}\right]\cdot \left[\begin{array}{c} \overset{••}{x}\\ \overset{••}{y} \end{array}\right]+\left[\begin{array}{cc} C_{xx} & 0\\ 0 & C_{yy} \end{array}\right]\cdot \left[\begin{array}{c} \overset{•}{x}\\ \overset{•}{y} \end{array}\right]+\\ \left[\begin{array}{cc} 2k_{1xx}+k_{3xx}+2k_{5xx}+k_{6xx}+k_{8xx} & 2k_{5xy}^{\alpha }+k_{6xy}^{\alpha }+k_{8xy}^{\alpha }\\ 2k_{5yx}^{\alpha }+k_{10yx}^{\alpha } & k_{4yy}+2k_{5yy}+k_{10yy}+2k_{2yy} \end{array}\right]\cdot \left[\begin{array}{c} x\\ y \end{array}\right]=\left[\begin{array}{c} F_{d}\\ F_{c,drive}=2m_{2}\prime \Omega_{z}\overset{•}{x} \end{array}\right] \end{array}$$

Assuming the electrostatic force *F_d_* = *F_e_* sin*ω_d_t*, the equation of driving displacement can be expressed below:

(9) $$x_{d}(t)=\frac{F_{e}/k_{xx}}{\sqrt{{\left[1-{\left(\frac{\omega_{d}}{\omega_{x}}\right)}^{2}\right]}^{2}+{\left(\frac{1}{Q_{drive}}\cdot \frac{\omega_{d}}{\omega_{x}}\right)}^{2}}}\sin(\omega_{d}t)$$

where *F_e_* is the amplitude of the electrostatic force, the *k_xx_* is the summation of 2*k*_1*xx*_, *k*_3*xx*_, 2*k*_5*xx*_, *k*_6*xx*_, *k*_8*xx*_. *ω_d_* is the frequency of driving voltage and *ω_x_* is the natural resonant frequency of Drive Mode, *Q_drive_* is the quality factor of the Drive Mode. *A_x,drive_* below is the amplitude of driving displacement when *ω_d_* ≈ *ω_x_*.

(10) $$A_{x,drive}=\frac{F_{e}Q_{drive}}{k_{xx}}=\frac{F_{e}Q_{drive}}{2k_{1xx}+k_{3xx}+2k_{5xx}+k_{6xx}+k_{8xx}}$$

In Equation (8), upon being steadily driven, even if there is no angular velocity input (Ω*_z_* = 0), the yaw sensing electrodes will be driven in direction y under the coupling term (*k*_5*yx*_*^α^ + k*_10*yx*_*^α^*)x, and quadrature error *y_Qerror,yaw_* between Drive Mode to Yaw Mode can be expressed below:

(11) $$\begin{array}{ll} y_{Qerror,yaw} & =\frac{-k_{yx}^{\alpha }A_{x,drive}}{(m_{2}\prime +m_{3})\sqrt{{\left(\omega_{y}^{2}-\omega_{x}^{2}\right)}^{2}+{\left(\frac{\omega_{x}\omega_{y}}{Q_{yaw}}\right)}^{2}}}\\ & =\frac{-(2k_{5yx}^{\alpha }+k_{10yx}^{\alpha })F_{e}Q_{drive}}{(2k_{1xx}+k_{3xx}+2k_{5xx}+k_{6xx}+k_{8xx})(m_{2}\prime +m_{3})\sqrt{{\left(\omega_{y}^{2}-\omega_{x}^{2}\right)}^{2}+{\left(\frac{\omega_{x}\omega_{y}}{Q_{yaw}}\right)}^{2}}} \end{array}$$

where *ω_y_* is the natural resonant frequency of Yaw Mode. *Q_yaw_* is the quality factor of the Yaw Mode. *k*_5*yx*_*^α^* and *k*_10*yx*_*^α^* are the coupling stiffness terms of decoupling beams Y_11_, Y_12_ and Y_7_, Y_8_, Y_9_, Y_10_ correspondingly. From the equation above, by eliminating the coupling stiffness terms 2*k*_5*yx*_*^α^ + k*_10*yx*_*^α^*, the quadrature error *y_Qerror,yaw_* can simultaneously be reduced. 

As mentioned in Equation (4), the terms of the coupling stiffness *k_xy_^α^(k_yx_^α^)* introduced by fabrication imperfection can be expressed as (*k_xx_ − k_yy_*)*α*. By reducing the equivalent angle of etching error α or conducting pre-compensation in the structural design on the key decoupling beams (Y_7_, Y_8_, Y_9_, Y_10_ and Y_11_, Y_12_), the quadrature error between Drive Mode to Yaw Mode can be reduced effectively.

Since the mechanical sensitivity of the Yaw Mode is linked with the input angular velocity Ω*_z_*, to simplify calculation, the coupling term (2*k*_5*yx*_ *+ k*_10*yx*_)x which leads to the quadrature error of Yaw Mode can be neglected in Equation (8). When the Drive Mode is in resonant state (*ω_x_* ≈ *ω_d_*), and the mechanical sensitivity *S_yaw_* of Drive Mode to Yaw Mode can be easily calculated and expressed below:

(12) $$\begin{array}{ll} S_{yaw} & =\frac{y_{out,yaw}}{\Omega_{z}}=\frac{\pi \cdot m_{2}\prime F_{e}\omega_{d}}{180k_{xx}k_{yy}}\frac{1}{\sqrt{{\left[1-{\left(\frac{\omega_{d}}{\omega_{x}}\right)}^{2}\right]}^{2}+{\left(\frac{1}{Q_{drive}}\cdot \frac{\omega_{d}}{\omega_{x}}\right)}^{2}}}\frac{1}{\sqrt{{\left[1-{\left(\frac{\omega_{d}}{\omega_{y}}\right)}^{2}\right]}^{2}+{\left(\frac{1}{Q_{yaw}}\cdot \frac{\omega_{d}}{\omega_{y}}\right)}^{2}}}\\ & =\frac{\pi \cdot m_{2}\prime F_{e}\omega_{d}Q_{drive}}{180(2k_{1xx}+k_{3xx}+2k_{5xx}+k_{6xx}+k_{8xx})(2k_{2yy}+k_{4yy}+2k_{5yy}+k_{10yy})}\frac{1}{\sqrt{{\left[1-{\left(\frac{\omega_{d}}{\omega_{y}}\right)}^{2}\right]}^{2}+{\left(\frac{1}{Q_{yaw}}\cdot \frac{\omega_{d}}{\omega_{y}}\right)}^{2}}} \end{array}$$

#### 3.1.2. Dynamics Analysis for Drive Mode to Pitch/Roll Mode

**Figure 7:**
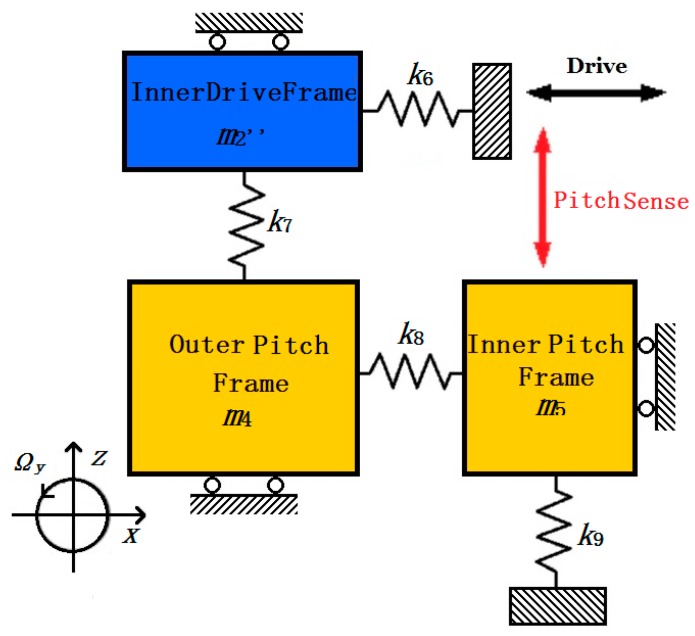
Simplified dynamic model for gyroscope in Pitch Mode.

The Pitch Sense Frame and Roll Sense Frame are symmetrically distributed in four directions of the MIMU. Since the whole structure of the MIMU is totally symmetrical (Figure 1 and Figure 3), the analyzing process for Pitch Mode is the same as Roll Mode. Take the Pitch Sense Frame into consideration, upon being driven steadily by the Drive Frame, the pitch sensing electrodes have an out-of-plane movement in direction z when input angular velocity Ω*_y_* applied on the MIMU. The simplified dynamic model of Pitch Mode is shown in Figure 7.

In Figure 7, the Inner Drive Frame (*m*_2_”) is driven together with the Big Frame (*m*_2_’) via decoupling beams Y_7_, Y_8_, Y_9_ and Y_10_ in Figure 3, so Pitch mode shares the same driving equation (Equation (6)) with the other sense mode as that in Yaw Mode.

*k*_6_ represents the summation of U-shaped driving beams D_9_, D_10_, D_11_, D_12_. Besides, *k*_7_ is the summation of Trampoline beams P_1_ and P_2_, and *k*_9_ is the summation of Trampoline beams P_3_ and P_4_. *k*_8_ represents the summation of four double U-shaped beams D_13_, D_14_, D_15_ and D_16_. The Outer Pitch Frame (*m*_4_) has two degrees of freedom and takes the Inner Pitch Frame (*m*_5_) move in direction *z* via double U-shaped beams (D_13_, D_14_, D_15_, D_16_). The dynamic equation of Pitch Mode in drive direction *x* and sense direction z can be written below:

(13) $$\begin{array}{l} \left[\begin{array}{cc} 2m_{1}+m_{2}+m_{4} & 0\\ 0 & m_{4}+m_{5} \end{array}\right]\left[\begin{array}{c} \overset{••}{x}\\ \overset{••}{z} \end{array}\right]+\left[\begin{array}{cc} C_{xx} & 0\\ 0 & C_{zz} \end{array}\right]\left[\begin{array}{c} \overset{•}{x}\\ \overset{•}{z} \end{array}\right]+\\ \left[\begin{array}{cc} 2k_{1xx}+k_{3xx}+2k_{5xx}+k_{6xx}+k_{8xx} & 0\\ k_{7zx}^{\theta }+k_{9zx}^{\theta } & k_{7zz}+k_{9zz} \end{array}\right]\left[\begin{array}{c} x\\ z \end{array}\right]=\left[\begin{array}{c} F_{d}\\ -2m_{4}\Omega_{y}\overset{•}{x} \end{array}\right] \end{array}$$

From analysis about the working principle in Section 2, the driving force in Drive Frame is the same as that in Inner Drive Frame, in other words, the Yaw Frame and Pitch/Roll Frame (Outer Pitch/Roll Frame and Inner Pitch/Roll Frame) are driven by the same driving force. Therefore, to simplify the calculation of mechanical sensitivity in Pitch/Roll Mode, the equation of driving movement in direction *x* (Equation (9)) can be reused.

The solving process of Pitch Mode is similar to that of Yaw Mode above, the mechanical sensitivity *S_pitch_* of Pitch Mode can be expressed below:

(14) $$\begin{array}{ll} S_{pitch} & =\frac{z_{out,pitch}}{\Omega_{y}}=\frac{\pi \cdot m_{4}F_{e}\omega_{d}}{180k_{xx}k_{zz}}\frac{1}{\sqrt{{\left[1-{\left(\frac{\omega_{d}}{\omega_{x}}\right)}^{2}\right]}^{2}+{\left(\frac{1}{Q_{drive}}\cdot \frac{\omega_{d}}{\omega_{x}}\right)}^{2}}}\frac{1}{\sqrt{{\left[1-{\left(\frac{\omega_{d}}{\omega_{z}}\right)}^{2}\right]}^{2}+{\left(\frac{1}{Q_{pitch}}\cdot \frac{\omega_{d}}{\omega_{z}}\right)}^{2}}}\\ & =\frac{\pi \cdot m_{4}F_{e}\omega_{d}Q_{drive}}{180(2k_{1xx}+k_{3xx}+2k_{5xx}+k_{6xx}+k_{8xx})(k_{7zz}+k_{9zz})}\frac{1}{\sqrt{{\left[1-{\left(\frac{\omega_{d}}{\omega_{z}}\right)}^{2}\right]}^{2}+{\left(\frac{1}{Q_{pitch}}\cdot \frac{\omega_{d}}{\omega_{z}}\right)}^{2}}} \end{array}$$

where *ω_z_* is the natural resonant frequency of Pitch/Roll Mode, *Q_pitch_* is the quality factor of the Pitch Mode. When the frequency of driving force *ω_d_* is equal to the natural resonant frequency of Drive Mode *ω_d_*, the mechanical sensitivity of Pitch/Roll Mode can be expressed in Equation (14). 

The source of the quadrature error *z_Qerror,pitch_* of Drive Mode to Pitch/Roll Mode is led by the coupling term *k_zx_^θ^* = (*k*_7*zx*_ *+ k*_9*zx*_)*θ*, where *θ* is the equivalent offset angle by etching error of decoupling beams P_1_, P_2_, P_3_ and P_4_ in Figure 4. The calculation process is the same as that in Equation (11) and expressed below.

(15) $$\begin{array}{ll} z_{Qerror,pitch} & =\frac{k_{zx}^{\theta }A_{x,d\mathrm{rive}}}{(m_{4}+m_{5})\sqrt{{\left(\omega_{z}^{2}-\omega_{x}^{2}\right)}^{2}+{\left(\frac{\omega_{x}\omega_{z}}{Q_{pitch}}\right)}^{2}}}\\ & =\frac{(k_{7zx}^{\theta }+k_{9zx}^{\theta })F_{e}Q_{drive}}{(2k_{1xx}+k_{3xx}+2k_{5xx}+k_{6xx}+k_{8xx})(m_{4}+m_{5})\sqrt{{\left(\omega_{z}^{2}-\omega_{x}^{2}\right)}^{2}+{\left(\frac{\omega_{x}\omega_{z}}{Q_{pitch}}\right)}^{2}}} \end{array}$$

By reducing the equivalent offset angle *θ* or making pre-compensation in the structural design on the key decoupling beams (P_1_, P_2_, P_3_ and P_4_), the quadrature error from Drive Mode to Pitch/Roll Mode can be reduced effectively.

### 3.2. Cross-Axis Error Analysis between Sense Modes

The sources of the total sense mode output can be divided into three parts: Coriolis effect output, quadrature error, and cross-axis error between sense modes [27,28]. However, the whole MIMU is totally symmetric in structure, whereas in separate structural part, since the decoupling beams are not symmetrically distributed and the existence of fabrication imperfection, there also exists a coupling effect between two different sense modes.

To calculate the cross-axis error between two different sense modes, one Sensing frame can be applied an assumed displacement, and by analyzing the coupling paths composed of various decoupling beams, the output of the other Sense Frame can be expressed. Since the Pitch Sense Frame and Roll Sense Frame are totally symmetric, the most significant cross-axis errors are those from Yaw Mode to Pitch/Roll Mode, and from Pitch/Roll Mode to Yaw Mode.

#### 3.2.1. Cross-Axis Error from Yaw Mode to Pitch Mode

**Figure 8:**
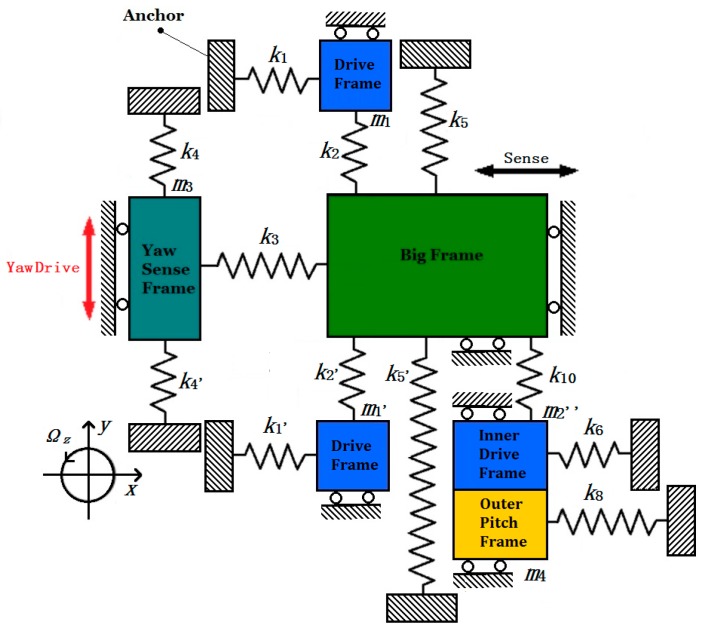
Simplified dynamic model of cross-axis error from Yaw Mode to Pitch Mode.

As is shown in Figure 3, if the Yaw Frame is driven in y axis by Coriolis force under the angular velocity input Ω*_z_*, the Inner Pitch Frame (*m*_5_) will be motionless in theory. Due to the existence of fabrication imperfection of the coupling beams (D_7_, D_8_, Y_7_, Y_8_, Y_9_, Y_10_, Y_11_, Y_12_), however, the Big Frame (*m*_2_’) together with the Inner Drive Frame (*m*_2_”) will be driven in turn by Yaw Frame, and further leads to the movement of Pitch Frame (*m*_4_ and *m*_5_), which will have an extra output signal called cross-axis error [23]. To analyze the cross-axis error, the dynamic model is established in Figure 8.

All the tabs of coupling beams correspond with those in the above figures. Assuming the “drive part” of the model is the yaw electrodes and angular input is Ω*_z_* only, and Yaw Sense Frame (*m*_3_) and Big Frame (*m*_2_’) move together in direction *y*, the displacement can be expressed by Equation (12) with mechanical sensitivity *S_yaw_*. Due to the existence of coupling terms *k*_5*xy*_*^α^*, Big Frame (*m*_2_’) as well as two Drive Frame (*m*_1_/*m*_1_’) have an extra displacement in direction *x*. By the transmission of decoupling beams Y_11_, Y_12_ and Y_7_, Y_8_, Y_9_, Y_10_ (*k*_5_/*k*_5_’ and *k*_10_), the extra displacement will be delivered to Inner Drive Frame (*m*_2_”), and further leads to the movement of Outer Pitch Frame (*m*_4_) and Inner Pitch Frame (*m*_5_).

The dynamic equation is similar to Equation (11). To express the driving displacement for Inner Drive Frame (*m*_2_”). The dynamic equations are written below:

(16) $$x_{s}(t)=\frac{(2k_{5xy}^{\alpha }+k_{10xy}^{\alpha })\Omega_{z}S_{yaw}}{(2m_{1}+m_{2}\prime +m_{2}\prime \prime )\sqrt{{\left(\omega_{x}^{2}-\omega_{d}^{2}\right)}^{2}+{\left(\frac{\omega_{x}\omega_{d}}{Q_{drive}}\right)}^{2}}}=\frac{(2k_{5xy}^{\alpha }+k_{10xy}^{\alpha })Q_{drive}\Omega_{z}S_{yaw}}{(2m_{1}+m_{2}\prime +m_{2}\prime \prime )\omega_{d}^{2}}=A_{sx}\Omega_{z}$$

where *ω_x_* is the equivalent resonant frequency for Inner Drive Mode. *Q_drive_* is the equivalent natural quality factors for Inner Drive Mode. *A_sx_* is the amplitude of the drive displacement for Inner Drive Frame (*m*_2_”). Actually, Inner Drive Mode shares the same resonant frequency and quality factor with Drive Mode. If Yaw Mode works in driving frequency *ω_d_*, Equation (16) can be simplified as above.

The calculation process for the output of the Pitch Mode is similar to that in Section 3.1.2, and the dynamic model is the same as Figure 7. Since the only input angular velocity is Ω*_z_*, the output of Pitch Mode is only from fabrication error of Pitch Mode, more specifically, from the terms of the coupling stiffness *k*_7*zx*_*^θ^ + k*_9*zx*_*^θ^*, and Equation (14) can be taken for referenced. So the cross-axis error from Yaw Mode to Pitch Mode can be expressed below:

(17) $$S_{yaw2pitch}=\frac{z_{out}}{\Omega_{z}}=\frac{k_{zx}^{\theta }A_{sx}}{(m_{4}+m_{5})\sqrt{{\left(\omega_{x}^{2}-\omega_{d}^{2}\right)}^{2}+{\left(\frac{\omega_{x}\omega_{d}}{Q_{pitch}}\right)}^{2}}}=\frac{(k_{7zx}^{\theta }+k_{9zx}^{\theta })(2k_{5xy}^{\alpha }+k_{10xy}^{\alpha })Q_{drive}S_{yaw}}{(2m_{1}+m_{2}\prime +m_{2}\prime \prime )(m_{4}+m_{5})\sqrt{{\left(\omega_{x}^{2}-\omega_{d}^{2}\right)}^{2}+{\left(\frac{\omega_{x}\omega_{z}}{Q_{pitch}}\right)}^{2}}\omega_{d}^{2}}$$

As is known, the smaller the cross-axis errors between two different sense modes, the better [29,30]. Since the main source of the cross-axis error from Yaw Mode to Pitch Mode is the coupling stiffness terms 2*k*_5*xy*_*^α^ + k*_9*zx*_*^α^* and *k*_7*zx*_*^θ^ + k*_9*zx*_*^θ^*, more specifically, the coupling stiffness terms of crab-leg beams Y_11_, Y_12_, U-shaped beams Y_7_, Y_8_, Y_9_, Y_10_, trampoline beams P_1_, P_2_, P_3_ and P_4_, that the equivalent fabrication angle *α* and *θ* matters most (Figure 4), should be decreased as much as possible to reduce the value of cross-axis error *S_yaw_*_2*pitch*_. 

#### 3.2.2. Cross-Axis Error from Pitch Mode to Yaw Mode

The mechanism of cross-axis error from Pitch Mode to Yaw Mode is similar with that from Yaw Mode to Pitch Mode. If the Inner Pitch Frame (*m*_5_) is driven in direction z by Coriolis force under the angular velocity input Ω*_y_*, the Yaw Sense Frame (*m*_3_) will be motionless in theory. Fabrication imperfection of the coupling beams (D_9_, D_10_, D_11_, D_12_, P_1_, P_2_) will lead to the movement of Outer Pitch Frame (*m*_4_) in direction *z*, together with the Inner Drive Frame (*m*_2_”) and Big Frame (*m*_2_’) in direction *x*, and further lead to the movement of Yaw Sense Frame (*m*_3_). The extra output signal of Yaw Frame is the cross-axis error from Pitch Mode to Yaw Mode.

Assuming the “drive part” of the model is the pitch electrodes. Due to the existence of *k*_7*zx*_*^θ^ + k*_9*zx*_*^θ^*, Inner Drive Frame (*m*_2_”) will have an extra movement, and further leads to the movement of Big Frame (*m*_2_’), under the decoupling beams Y_7_, Y_8_, Y_9_, Y_10_ (*k*_10_). Then, Yaw Sense Frame (*m*_3_) will further be driven by Y_11_, Y_12_ (*k*_5*yx*_*^α^/k*_5*yx*_*^α^’*). Dynamic model in Figure 5 can be taken for reference and the dynamic equations are written below, from which the equivalent drive displacement for Inner Drive frame together with the Big Frame can be calculated.

(18) $$x_{s}(t)=\frac{(k_{7xz}^{\theta }+k_{9xz}^{\theta })\Omega_{y}S_{pitch}}{(m_{2}\prime \prime +m_{4})\sqrt{{\left(\omega_{x}^{2}-\omega_{d}^{2}\right)}^{2}+{\left(\frac{\omega_{x}\omega_{d}}{Q_{drive}}\right)}^{2}}}=\frac{(k_{7xz}^{\theta }+k_{9xz}^{\theta })Q_{drive}\Omega_{y}S_{pitch}}{(m_{2}\prime \prime +m_{4})\omega_{d}^{2}}=A_{sx}\Omega_{y}$$

where *ω_x_*, *Q_sx_*, *A_sx_* and *ω_d_* are the same parameters as those in Equation (16). Therefore, Equation (18) can be simplified as above.

The calculation process for the output of the Yaw Mode is similar to that in Section 3.1.1, and the dynamic model is the same as Figure 6. Since the only input angular is Ω*_y_*, the output of Yaw Mode is only from fabrication error of decoupling beams Y_11_, Y_12_ and Y_7_, Y_8_, Y_9_, Y_10_, more specifically, from the terms of the coupling stiffness 2*k*_5*yx*_*^α^ + k*_10*yx*_*^α^*, and Equation (11) can be taken for reference. So the cross-axis error from Pitch Mode to Yaw Mode can be expressed below:

(19) $$S_{pitch2yaw}=\frac{y_{out}}{\Omega_{y}}=\frac{(2k_{5yx}^{\alpha }+k_{10yx}^{\alpha })A_{sx}}{(m_{2}\prime +m_{3})\sqrt{{\left(\omega_{y}^{2}-\omega_{x}^{2}\right)}^{2}+{\left(\frac{\omega_{x}\omega_{y}}{Q_{yaw}}\right)}^{2}}}=\frac{(2k_{5yx}^{\alpha }+k_{10yx}^{\alpha })(k_{7xz}^{\theta }+k_{9xz}^{\theta })Q_{drive}S_{pitch}}{(m_{2}\prime +m_{3})(m_{2}\prime \prime +m_{4})\sqrt{{\left(\omega_{y}^{2}-\omega_{x}^{2}\right)}^{2}+{\left(\frac{\omega_{x}\omega_{y}}{Q_{yaw}}\right)}^{2}}\omega_{d}^{2}}$$

The main source of the cross-axis error from Pitch Mode to Yaw Mode is the coupling stiffness terms 2*k*_5*yx*_*^α^ + k*_10*yx*_*^α^* and *k*_7*zx*_*^θ^ + k*_9*zx*_*^θ^*, more specifically, the coupling stiffness terms of the crab-leg beams Y_11_/Y_12_ and Y_7_, Y_8_, Y_9_, Y_10_, linked with the Big Frame (*m*_2_’), and trampoline beams P_1_, P_2_, P_3_ and P_4_. The equivalent fabrication angle *α*, and *θ* (Figure 4), should be decreased as much as possible to reduce the value of cross-axis error *S_pitch2yaw_.*

**Figure 9:**
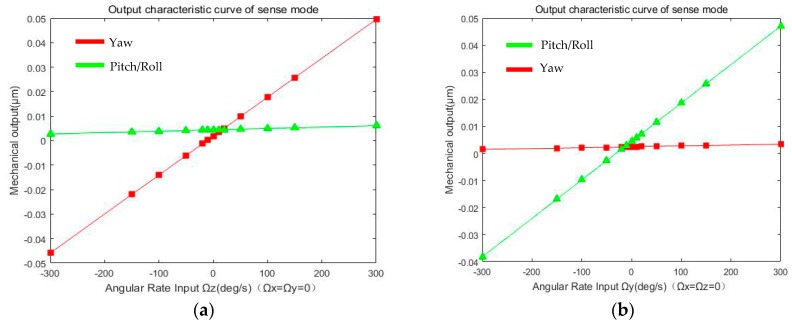
Simulation of mechanical output for sense modes: (**a**) Yaw Mode with angular rate input Ω*_z_*; (**b**) Pitch (Roll) Mode with angular rate input Ω*_y_* (Ω*_x_*).

Equation (20) shows the mechanical output of three sense modes, when the Drive Mode is in resonant state and been make mode-matching with sense modes for high sensitivity. Figure 9 shows the simulation of the mechanical output for sense modes.

(20) $$\left[\begin{array}{c} y_{out}^{yaw}\\ z_{out}^{pitch}\\ z_{out}^{roll} \end{array}\right]=\left[\begin{array}{ccc} S_{drive2yaw} & S_{pitch2yaw} & S_{roll2yaw}\\ S_{yaw2pitch} & S_{drive2pitch} & S_{roll2pitch}\\ S_{yaw2roll} & S_{pitch2roll} & S_{drive2roll} \end{array}\right]\left[\begin{array}{c} \Omega_{z}\\ \Omega_{y}\\ \Omega_{x} \end{array}\right]+\left[\begin{array}{c} y_{Qerror,yaw}^{}\\ z_{Qerror,pitch}\\ z_{Qerror,roll} \end{array}\right]=\left[\begin{array}{ccc} S_{yaw} & S_{pitch2yaw} & S_{roll2yaw}\\ S_{yaw2pitch} & S_{pitch} & 0\\ S_{yaw2roll} & 0 & S_{roll} \end{array}\right]\left[\begin{array}{c} \Omega_{z}\\ \Omega_{y}\\ \Omega_{x} \end{array}\right]+\left[\begin{array}{c} y_{Qerror,yaw}\\ z_{Qerror,pitch}\\ z_{Qerror,roll} \end{array}\right]$$

The *S_yaw_*, *S_pitch_*, *S_roll_* represent the mechanical sensitivity of three sense modes; The *S_yaw_*_2*pitch*_ (*S_yaw_*_2*roll*_), *S_pitch_*_2*yaw*_ (*S_roll_*_2*yaw*_), *S_roll_*_2*pitch*_ (*S_pitch_*_2*roll*_) represent the cross-axis error from one sense mode to the other mode, respectively. In addition, the *y_Qerror,yaw_*, *z_Qerror,pitch_*, *z_Qerror,roll_* are the quadrature error corresponding to the sense modes.

Cross-axis error from Pitch/Roll Mode to Yaw mode is about 5.6 × 10^−6^ μm/°/s, and the mechanical sensitivity of Yaw Mode is about 1.59 × 10^−4^ μm/°/s, with quality factor (*Q_yaw_*) of in-plane movement in vacuum about 300. Quadrature error of Yaw Mode is 1.849 × 10^−3^ μm with fabrication error *α* = 0.1° of decoupling beams *k*_5_/*k*_5_’ (Y_11_/Y_12_) and *k*_10_ (Y_7_, Y_8_, Y_9_, Y_10_). Likewise, cross-axis error from Yaw Mode to Pitch/Roll Mode is about 2.4 × 10^−6^ μm/°/s, and the mechanical sensitivity of Pitch/Roll Mode is about 1.42 × 10^−4^ μm/°/s, with quality factor (*Q_pitch_/Q_roll_*) of out-of-plane movement in vacuum about 1000. Quadrature error of Pitch/Roll Mode is 4.397 × 10^−3^ μm with fabrication error *θ* = 0.1° of decoupling beams *k*_7_ (P_1_/P_2_) and *k*_9_ (P_3_/P_4_). Summary of all the parameters above is shown in Table 4.

## 4. Fabrication Process

**Figure 10:**
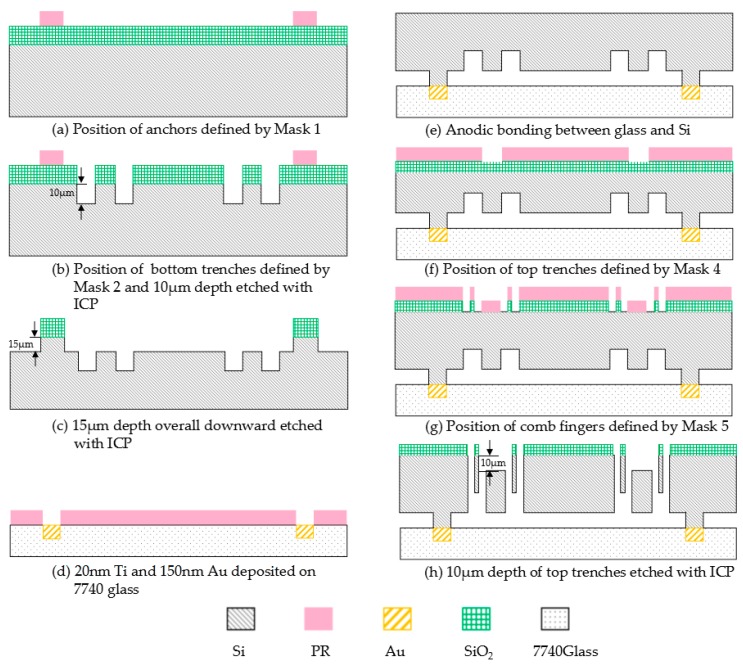
Main steps of the fabrication process.

The six-DOF MIMU with anchors, decoupling beams and driving and sensing combs can be fabricated using 5 masks by silicon on glass (SOG) process, including silicon/glass wafer bonding and deep reactive ion etching (DRIE). The total die size of the MIMU is 10,000 μm × 10,000 μm and the thickness of the structure is designed to 60 μm. The fabrication is supported by Suzhou Institute of Nano-Tech and Nano-Bionics (SINANO), China. The main steps of the fabrication process are shown in Figure 10.

Firstly, the position of all the anchors is defined by Mask 1, which is the lithography mask used for photolithography in the lithography process with positive photoresist AZ6130 (Suzhou Ruihong Electronic Chemical Co., Ltd., Suzhou, China). Hexamethyldisilazane (HMDS) for pretreatment can be adopted to increase the adhesion between the photoresist and silicon wafer before spin coating the photoresist. RZX-3038 (developer for positive photoresist, Suzhou Ruihong Electronic Chemical Co., Ltd., Suzhou, China) is used for about 45 s to 60 s in lithography development. The 500 nm thick thermally grown SiO_2_ is worked as sacrificial layer. Reactive Ion Etching (RIE) is adopted to etch the SiO_2_ and exposes the underneath silicon for the MIMU structure, including decoupling beams, comb fingers and proof masses etc. (Figure 10a).

Secondly, the photoresist AZ6130 is used to define the position of bottom trenches for out-of-plane comb fingers by Mask 2 with lithography process. Photoresist must be maintained as a mask layer and the bottom trenches are etched 10 μm depth by ICP (inductive coupled plasma) etching (Figure 10b).

Thirdly, all the photoresist should be removed, and etched overall downward for 15 μm depth by ICP etching once again to form the anchors (Figure 10c). Too fast etching rate may cause lateral etching and over-etching, while too slow etching rate can increase roughness of the comb fingers and reduce the fabrication quality. After many attempts, the rate of silicon etching for ICP is set from 500 nm/min to 600 nm/min by controlling the power of STS. Then, all the SiO_2_ covering on the anchors for anodic bonding should be removed, with wet etching in buffered oxide etch (BOE) solution (49%HF: 40%NH_4_F = 1:5). 

Meanwhile, 20 nm Ti and 150 nm Au are successively deposited on the Pyrex 7740 glass (CORNING, Corning, NY, USA) by Mask 3 with DC reactive magnetic sputtering and lift-off process soaking in the acetone solution (Figure 10d). Anodic bonding between Pyrex 7740 glass and anchors involves alignment precision ensured by alignment marks on Mask 1 and Mask 3. Mechanical polishing is performed afterwards. Thickness of the silicon wafer should be thinned to 75 μm, including structure (60 μm) and anchors (15 μm) (Figure 10e). The top side of the wafer is covered by SiO_2_ deposited with plasma enhanced chemical vapour deposition (PECVD) for sacrificial layer for next step, the thickness of SiO_2_ can be chosen from 500 nm to 600 nm.

Next, photoresist is spin-coated to define the position of top trenches for out-of-plane comb fingers by Mask 4 with lithography process and RIE, for which the covering accuracy and alignment precision of top and bottom trenches are of crucial importance (Figure 10f). The precision is ensured by registration marks on Mask 2 and Mask 4. All the residual photoresist must be removed then. 

**Figure 11:**
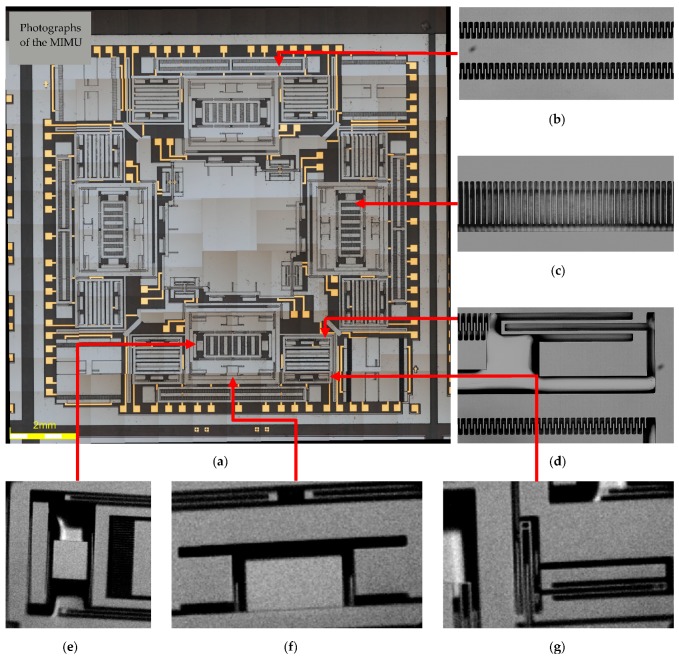
Photographs of the MIMU structure: (**a**) Whole structure of the MIMU; (**b**) Detailed comb fingers of the driving electrodes in Drive Frame; (**c**) Detailed comb fingers of the pitch electrodes in Pitch Frame; (**d**) The anchors and U-shaped coupling beams of Drive Frame; (**e**) Trampoline beams in Inner-Roll Frame; (**f**) Trampoline beams in Outer-Roll Frame; (**g**) Detailed decoupling beams between Big Frame and Outer-Roll Frame.

Then, new photoresist is spin-coated to define the position of the gap of comb fingers by Mask 5 with lithography process and RIE (Figure 10g). It is vital for the whole fabrication process in this step because the width of comb fingers are designed to 5 μm and the gap between the fingers is 3 μm (Figure 11). Since fabrication error has been accumulated in the above steps, to ensure the design precision, 1 or 2 microns can be adjusted in lithography process. The photoresist must be retained as mask layer and etch the silicon to release the structure by ICP etching. 

Finally, all the photoresist should be removed and form the top trenches by ICP etching once again. Etching depth is 10 μm (Figure 10h). Residual SiO_2_ should be removed by BOE solution (49% HF: 40% NH_4_F = 1:5) for several hours.

Since the fabrication error accumulates during several steps of lithography alignment and silicon etching, to ensure the design accuracy, alignment of negative trenches (Figure 10b) and positive trenches (Figure 10f) is of great importance. Furthermore, if the bonding strength between Pyrex 7740 glass and silicon structure is less enough than demanded, misplacement of structure on the glass may take place during the process of thinning and mechanical polishing (Figure 10e). Figure 11 shows the photographs of the MIMU structure and its detailed parts. Figure 11a shows the whole structure of the MIMU; Figure 11b shows the comb fingers of the driving electrodes with in-plane movement; Figure 11c shows the comb fingers of the pitch electrodes with out-of-plane movement; Figure 11d shows the anchors and U-shaped coupling beams of the Drive Frame; Figure 11e and Figure 11f show the Trampoline beams in roll electrodes; Figure 11g show the decoupling beams between Big Frame and Outer-Roll Frame.

The fabrication process of the presented MIMU is done firstly by etching the bottom trenches, and anchors afterwards. Limited by the ICP etching, the edge and corner of the trenches may be much rougher than the design, because both the bottom trenches and anchors are etched overall downward. To reduce surface roughness, the sequence of the ICP etching for bottom trenches and anchors can be exchanged. The anchors can be firstly etched by ICP for 15 μm depth, and then photoresist is spin-coated to define the position of bottom trenches in lithography process. 10 μm depth of Si should be etched by ICP to form the bottom trenches.

Protected by the photoresist, roughness of the edge and corner in this fabrication process, which is applicable to the formation of the top trenches, is much smaller than the former one. 

**Figure 12:**
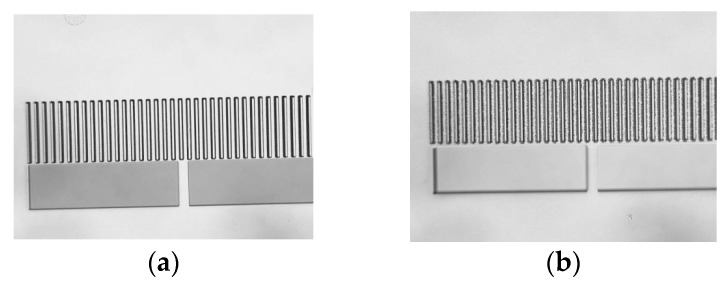
(**a**) Bottom trenches first and anchors afterwards in ICP etching process for smooth edge; (**b**) Anchors first, and bottom trenches afterwards with the protection of photoresist in ICP etching process for rough edge.

Figure 12a,b are the comparison of roughness between the two fabrication processes.

**Figure 13:**
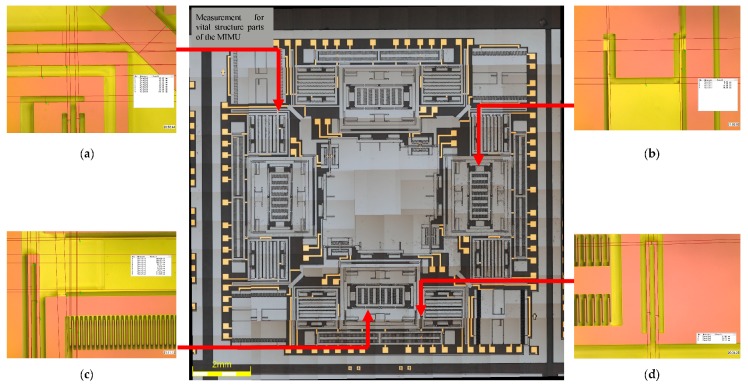
Measurement for vital structure parts of the MIMU. (**a**) Crab-leg beams and U-shaped beams linking the Big Frame; (**b**) Trampoline beams in Outer-Pitch Frame; (**c**) Double U-shaped beams in Inner-Roll Frame; (**d**) U-shaped beams linking Drive Frame and Big Frame.

As analyzed in Section 3.1, fabrication imperfection can introduce the fabrication error and cause an influence on the stiffness of some of the decoupling beams (such as U-shaped beam Y_7_, Y_8_, Y_9_ and Y_10_), which is the main source of the quadrature error of different sense modes. By measuring the size of the MIMU structure in microscope, fabrication quality and fabrication error can be acquired. Figure 13 shows the measurement for some vital structural parts of the MIMU. In order to improve the contrast of the structure and make it clear to express the vital structure parts of the MIMU in the photograph by microscope, the SiO_2_ covered on the structure with pink color is not disposed by BOE. Table 5 shows both the designed and actual parameters of the vital structure of the MIMU.

## 5. Experimental Results

**Figure 14:**
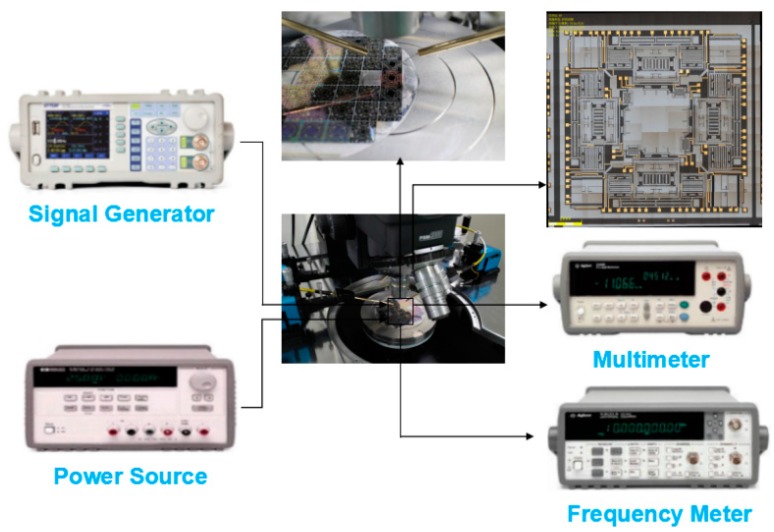
Experimental setup for mode detection and quality factors in air.

**Figure 15:**
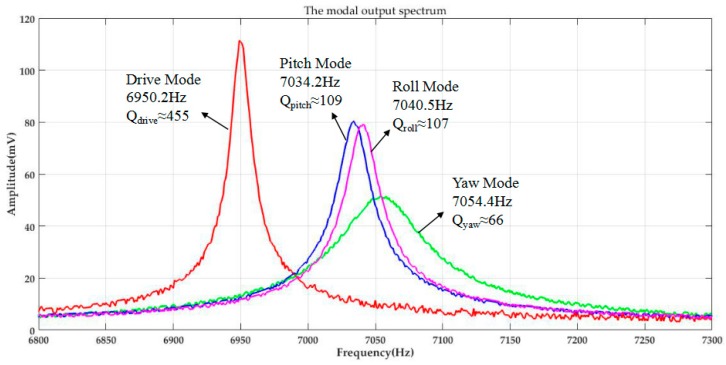
The output spectrum for resonant frequencies and quality factors of four working modes.

The fabricated MIMU is assembled onto the probe station without being vacuum-sealed. Figure 14 shows the experimental setup for mode detection and quality factors of Drive Mode, Yaw Mode, Pitch Mode and Roll Mode in air. The modal spectrum indicating resonant frequencies and quality factors of corresponding mode is shown in Figure 15.

The modal output spectrum figure shows the resonant frequencies and quality factors of corresponding mode in air: resonant frequency of the Drive Mode is 6950.2 Hz, with the quality factor *Q_drive_* about 455; resonant frequency of the Yaw Mode is 7054.4 Hz with the quality factor *Q_yaw_* about 66, whose frequency split is about 104.2 Hz relative to the Drive Mode; the Pitch Mode and Roll Mode resonant frequencies are 7034.2 Hz and 7040.5 Hz with quality factor *Q_pitch_* for 109 and *Q_roll_* for 107, whose frequency split are about 84 Hz and 90.3 Hz. Although the Pitch Frame and Roll Frame of the MIMU are totally symmetrical in design, there still exists mismatching in actual fabrication process, whereas the difference is in an acceptable range. Vacuum sealed packaging and temperature prediction and controlling of circuit [31] can enhance the quality factor and further improve the performance of the whole device, which is the research emphasis in future work. 

## 6. Conclusions

A novel fully decoupled micro inertial measurement unit (MIMU) mainly involving three-axis micro-gyroscope is designed and fully characterized. A variety of elaborated decoupling beams are designed and analyzed to acquire full decoupling between drive mode and sense modes, under the existence of fabrication error (for example about 0.1°) of the key decoupling beams. Based on the detailed analyses of dynamic models with decoupling beams stiffness, the mechanical sensitivity, cross-axis errors and quadrature error of sense modes are presented, whose values are simulated by MATLAB R2016b (MathWorks, Natick, MA, USA). The whole fabrication process is contained afterwards, the covering accuracy and alignment precision of top and bottom trenches are of crucial importance that can influence the quality factor of the whole MIMU. The method for reducing the edge roughness of comb fingers is to change the etching order of trenches and anchors (or structure for being released). Finally, experiments for the output of four working modes (drive, yaw, pitch and roll) are performed, whose resonant frequencies are 6950.2 Hz, 7054.4 Hz, 7034.2 Hz and 7040.5 Hz. In addition, quality factors of each mode detected by probe station are 455, 66, 109 and 107 respectively in air. The results demonstrate that the proposed structure has exhibited potential to achieve good performance.

To further obtain higher working performance, vacuum packaging with silicon-on-insulator (SOI) for reducing the air damping can be considered in the following study. Digitization for the test circuit should also be taken into account. However, above all, improving the fabrication technique, for instance improving the precision of the comb fingers and reducing the fabrication error of decoupling beams, is the most urgent matter in the future research. 

## Figures and Tables

**Table 1:** Summary of part number and function description of beams & electrodes in three modes.

Function Description			Part Number
Gyroscope Unit	Drive Mode	U-shaped beam	D_1_, D_2_, D_3_, D_4_
			D_1_’, D_2_’, D_3_’, D_4_’
			D_5_, D_6_, D_7_, D_8_
		Drive electrodes	D-a, D-b
	Yaw Mode	U-shaped beam	Y_1_, Y_2_, Y_3_, Y_4_, Y_5_, Y_6_
		Crab-leg beam	Y_11_, Y_12_
		Yaw electrodes	Y-a
	Pitch/Roll Mode	U-shaped beam	D_9_, D_10_, D_11_, D_12_
		Double U-shaped beam	D_13_, D_14_, D_15_, D_16_
		Trampoline beam	P_1_, P_2_, P_3_, P_4_
		Pitch electrodes	P-a, P-b
		Roll electrodes	R-a, R-b
Accelerometer Unit	Acceleration for direction *x*/*y*		Acc-*xy*
	Acceleration for direction *z*		Acc-*z*

**Table 2:** Part of the structural dimensions with fabrication error (*α = θ =* 0.1°).

Tabs	Summation of Coupling Beams	*K_xx_* [N/m]	*k_yy_* [N/m]	Coupling Terms [N/m]
*k*_1_/*k*_1_’	D_1_, D_2_, D_3_, D_4_/D_1_’, D_2_’, D_3_’, D_4_’	49.6576	1950	190.0342
*k*_2_/*k*_2_’	Y_3_/Y_4_	1860.7	43.2814	181.7419
*k*_3_	D_5_, D_6_, D_7_, D_8_	53.3203	1998	194.4680
*k*_4_	Y_1_, Y_2_, Y_5_, Y_6_	1603.6	27.9724	157.5628
*k*_5_/*k*_5_’	Y_11_/Y_12_	53.4492	96.8923	39.6541
*k*_6_	D_9_, D_10_, D_11_, D_12_	49.6576	1950	380.0684
*k*_7_	P_1_, P_2_	2165.9	101.0704 (*k_zz_*)	112.4212
*k*_8_	D_13_, D_14_, D_15_, D_16_	71.2309	3482.1	341.0869
*k*_9_	P_3_, P_4_	2163.5 (*k_yy_*)	203.9288 (*k_zz_*)	115.5196
*k*_10_	Y_7_, Y_8_, Y_9_, Y_10_	1669.5	31.4864	163.8014

**Table 3:** Weight of different masses in a quarter of the structure.

Parameter and Variable Name	Symbol	Value [Unit]
Drive Frame	*m*_1_/*m*_1_’	1.04508 × 10^−4^ g
Big Frame	*m*_2_’	1.6699392 × 10^−4^ g
Inner Drive Frame	*m*_2_”	3.456 × 10^−5^ g
Mass in Yaw Mode	*m*_3_	1.154208 × 10^−4^ g/2.8224 × 10^−5^ g
Outer Pitch/Roll Frame	*m*_4_	2.5531 × 10^−4^ g
Inner Pitch/Roll Frame	*m*_5_	7.503 × 10^−5^ g

**Table 4:** Summary for mechanical sensitivity, cross-axis errors and quadrature error of sense modes.

Mechanical Sensitivity (μm/°/s)		Cross-Axis Error (μm/°/s)	Quadrature Error (μm)	Capacity Sensitivity (F/°/s)
Yaw Mode	*S_yaw_* = 1.59 × 10^−4^ *Q_yaw_* ≈ 300	*S_pitch2yaw_/S_pitch2yaw_* = 5.6 × 10^−6^	*y_Qerror,yaw_* = 1.849 × 10^−3^ (*α* = 0.1°)	*S_cyaw_* = 8.56 × 10^−17^
Pitch/Roll Mode	*S_pitch_/S_roll_* = 1.42 × 10^−4^ *Q_pitch_* ≈ 1000	*S_yaw2pitch_/S_roll2pitch_* = 2.4 × 10^−6^	*z_Qerror,pitch_/z_Qerror,roll_* = 4.397 × 10^−3^ (*θ* = 0.1°)	*S_cpitch_/S_croll_* = 3.43 × 10^−17^

**Table 5:** Measurement for parameters of vital decoupling beams of the MIMU.

Tabs	Dimensions	Design Size (μm)	Actual Measured Size (μm)		Fabrication Rotation Error (°)
U-shaped beam (D_5_–D_8_/D_1_–D_4_/ Y_3_, Y_4_/Y_7_–Y_10_/ Y_1_, Y_2_, Y_5_, Y_6_)	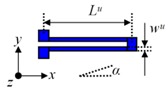	*L^u^* = 400/410 /430/480 /500 *w^u^*= 10		*L^u^* = 400.21/409.58 /430.54/478.98 /500.16 *w^u^* = 11.09	α = 0.13–0.53
Crab-leg beam (Y_11_, Y_12_)	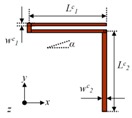	*L^c^*_1_ = 2040 *L^c^*_2_ = 1622.5 *w^c^*_1_ = 45 *w^c^*_2_ = 45		*L^c^*_1_ = 2038.37 *L^c^*_2_ = 1623.29 *w^c^*_1_ = 44.27 *w^c^*_2_ = 44.34	α = 0.15–0.55
Double U-shaped beam (D_13_–D_16_)	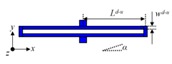	*L^d−u^* = 460 *w^d−u^* = 10		*L^d−u^* = 460.74 *w^d−u^* = 10.32	α = 0.11–0.36
Trampoline beams (P_1_, P_2_/P_3_, P_4_)	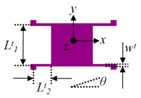	*L^t^*_1_ = 176/250 *L^t^*_2_ = 170 *w^t^* = 8		*L^t^*_1_ = 175.32/248.87 *L^t^*_2_ = 169.46 *w^t^* = 7.83	*θ* = 0.11–0.24
